# Plant Derived Natural Products against *Pseudomonas aeruginosa* and *Staphylococcus aureus*: Antibiofilm Activity and Molecular Mechanisms

**DOI:** 10.3390/molecules25215024

**Published:** 2020-10-29

**Authors:** Francesca Guzzo, Monica Scognamiglio, Antonio Fiorentino, Elisabetta Buommino, Brigida D’Abrosca

**Affiliations:** 1 Dipartimento di Scienze e Tecnologie Ambientali Biologiche e Farmaceutiche–DiSTABiF, Università degli Studi della Campania “Luigi Vanvitelli”, via Vivaldi 43, I-81100 Caserta, Italy; francesca.guzzo@unicampania.it (F.G.); monica.scognamiglio@unicampania.it (M.S.); antonio.fiorentino@unicampania.it (A.F.); 2 Dipartimento di Biotecnologia Marina, Stazione Zoologica Anton Dohrn, Villa Comunale, 80121 Naples, Italy; 3 Dipartimento di Farmacia, Università degli Studi di Napoli “Federico II”, Via Domenico Montesano 49, 80131 Napoli, Italy; elisabetta.buommino@unina.it

**Keywords:** plant-derived natural products, terpenes, flavonoids, antibiotic-resistance, biofilm, quorum sensing, *Pseudomonas aeruginosa*, *Staphylococcus aureus*

## Abstract

Bacteria are social organisms able to build complex structures, such as biofilms, that are highly organized surface-associated communities of microorganisms, encased within a self- produced extracellular matrix. Biofilm is commonly associated with many health problems since its formation increases resistance to antibiotics and antimicrobial agents, as in the case of *Pseudomonas aeruginosa* and *Staphylococcus aureus*, two human pathogens causing major concern. *P. aeruginosa* is responsible for severe nosocomial infections, the most frequent of which is ventilator-associated pneumonia, while *S. aureus* causes several problems, like skin infections, septic arthritis, and endocarditis, to name just a few. Literature data suggest that natural products from plants, bacteria, fungi, and marine organisms have proven to be effective as anti-biofilm agents, inhibiting the formation of the polymer matrix, suppressing cell adhesion and attachment, and decreasing the virulence factors’ production, thereby blocking the quorum sensing network. Here, we focus on plant derived chemicals, and provide an updated literature review on the anti-biofilm properties of terpenes, flavonoids, alkaloids, and phenolic compounds. Moreover, whenever information is available, we also report the mechanisms of action.

## 1. Introduction

The classical concept of microorganisms as solitary entities was revised when it appeared clear that bacteria, as well as fungi, are social organisms able to build complex communities, like biofilms. This condition facilitates survival in adverse conditions, allowing microorganisms to grow and colonize host tissues or inert surfaces, including implants and urinary catheters [1], with adverse effects on human health. Therefore, a great effort is needed to find new drugs able to counteract this phenomenon, with natural products in the hotspot as possible promising candidates.

Biofilm has a very complex architecture. Microorganisms embedded in the biofilm are not simply attached to a surface; complex molecular signals, inducing the spatial and temporal reorganization of planktonic cells, are activated in response to environmental stimuli. As a consequence, bacteria in the biofilm show altered expression of surface molecules, nutrient utilization, and virulence factors, along with increased stress resistance. These factors allow their survival in unfavorable environments, but also resistance to antimicrobial compounds and evasion of host immunity [2,3]. Therefore, it is important for the host to eliminate bacteria before biofilm is organized.

Even more complex is the situation in which biofilms are polymicrobial [1]. Co-infecting species in the polymicrobial biofilm can aggravate the severity of the disease, complicating the choice of antibiotic therapy, with a consequent delay in host recovery. One example of a polymicrobial infection is given by *Pseudomonas aeruginosa* and *Staphylococcus aureus*. They separately colonize different niches (soil and water in the former, and the respiratory tract and skin in the latter), but can cause chronic wound infections that are resistant to conventional antimicrobial therapy [4].

*P. aeruginosa* is one of the most common pathogens in nosocomial and ventilator-associated pneumonia, cystic fibrosis (CF), meningitis, abscess, soft tissue and urinary tract infections, infection of the cornea, and erythema of conjunctiva. In addition, it can cause catheter-associated and chronic lung infections in immunocompromised patients [5]. Considering its ability to form biofilm on medical devices and to take advantage on the host with an altered normal flora, due to administration of broad-spectrum antibiotics, *P. aeruginosa* has induced researchers to study and develop new therapeutic strategies.

On the other hand, *S. aureus* is a leading cause of both community- and hospital-acquired infections associated with high morbidity and mortality, due also to the emergence of multi-drug resistant strains such as MRSA (Methicillin-Resistant *S. aureus*) [6]. Although it is found in the environment, and can be part of normal human flora (colonizing the skin and mucous membranes of healthiest individuals), if it finds the conditions to enter the bloodstream or internal tissues it may be responsible for different, potentially serious, infections.

Cystic fibrosis, otitis media, periodontitis, urinary tract infections, and osteomyelitis, are all polymicrobial [7]. For example, *P. aeruginosa* and *S. aureus* are often found to coinfect the lungs of patients with CF, and alginate overproduction may be an important factor driving *P. aeruginosa* coinfection with *S. aureus* [8,9].

Multiple and complex are the mechanisms that lead to acquired resistance to antibiotics in microorganisms. Among these, reduced permeability through the cell membrane, modification of the molecular target, increased efflux pump expression, and degradation of the antibiotics are all important factors that reduce drug efficacy on free planktonic cells. In addition, in bacterial biofilm, other modifications are induced, like decreased growth rates and metabolism, and induction of cell biofilm-specific phenotypes, known as persister cells [10]. As a consequence, antibiotics active against planktonic cells can result in being ineffective on sessile/dormant cells. Many studies have attempted to understand the molecular mechanism underlying antibiotic inefficacy in sessile bacteria. The reckless use of antibiotics has led to the development of multidrug resistant microorganisms, due to the selective pressure exerted on their survivability; therefore, gene expression modulation of microorganism virulence factors, rather than killing them, has been widely explored. Natural products are mainly directed at inhibiting bacterial growth or at reducing their pathogenicity, acting on specific genes that control important virulence factors [11]. In addition, several natural compounds have also been explored for their properties as quorum sensing (QS) inhibitors [12].

Plants, microorganisms, as well as marine organisms, represent an inestimable source of anti-biofilm agents. Examples of these compounds are styrylpyrones and quinic acid derivatives from the polar extract of *Helichrysum italicum* (active against *P. aeruginosa* [13]), pholretin, (specifically reducing enterohemorrhagic *Escherichia coli* O157:H7 (EHEC) biofilm formation [14]), or alkaloids from marine sponges (active against gram-positive and gram-negative bacteria [15]).

Many studies have been conducted with the aim of discovering novel antimicrobial and anti-biofilm agents [16]. Anti-biofilm intervention can be aimed at mechanical eradication/destruction (modification of surface properties of the biofilm carrier and mechanical stability of the biofilm, application of hydrolytic enzymes disrupting its structure and composition, and others) as well as acting on the regulatory system of its formation (QS and virulence factors) [17].

The aim of this review is to examine the most recent literature in the field of plant-derived natural products as potential novel anti-biofilm agents against *P. aeruginosa* and *S. aureus*. We briefly summarize the mechanisms of biofilm formation and QS for both bacteria objects of the present review. The anti-biofilm effects of natural products, mainly relying on the inhibition of formation of the polymer matrix, suppression of cell adhesion and attachment, and decrease of virulence factor production, thereby blocking QS network, are summarized. Furthermore, as mentioned above, biofilm formation is driven by sophisticated regulatory mechanisms, involving events both at single-cell level and at cell population level. Thus, in this paper we will discuss the molecular mechanisms associated with the anti-biofilm effects of terpene, flavonoids, alkaloids, and phenolic compounds.

## 2. Quorum Sensing Mechanism

Among the regulatory mechanisms that ensure timely adaptation of microorganisms to the environment, QS is the most studied since it plays a critical role in the formation of biofilm and its surrounding extracellular polymeric substance (EPS). The latter is important to keeping the basic architecture of a biofilm matrix, and forms the defense shield for bacteria inside the biofilm [18]. EPS quantification can directly correlate with the extent of biofilm formation. The EPS protects bacteria from the antimicrobial activity of antibiotics. It comprises 50–90% of the total organic mass of the biofilm and contains exopolysaccharides, extracellular DNA (eDNA), proteins, lipids, and humic substances [19]. Biofilm is not just a barrier to avoid the deleterious effect of antibiotics but also increases bacteria pathogenicity, through the activation of genes that control their virulence. QS is a cell-to-cell communication, depending on density population, and it is differently gene-controlled in gram-negative and gram-positive bacteria, as well as in fungi. It controls the expression of important bacterial genes that encode for virulence factors [20,21]. The QS system is mediated by autoinducers (AIs), identified as oligopeptides and acylated homoserine lactones (AHLs) in gram-positive and gram-negative bacteria, respectively.

### 2.1. QS Molecular Signaling Network of Gram-Negative Bacteria

**Figure 1:**
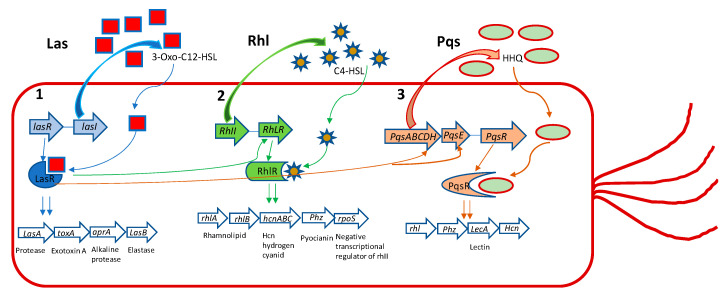
The three major *Pseudomonas aeruginosa* QS systems with their main effects. (**1**) LasI produces 3-oxo-C12-HSL, which acts on LasR. This leads to induction of *aprA*, *lasA*, *las B*, and *toxA* genes and other virulence genes that are under its regulation. (**2**) RhlI produces C4-HSL, that acts on RhlR, which induces *phz*, *lasA*, *rpoS*, *lasB*, *rhlAB*, and *hcnABC* gene expression. (**3**) PqsABCDH produces HHQ that acts on PqsR, regulating the gene expression of *LecA*, *Phz*, *Hcn*, and *rhl*. Additionally, LasR positively regulates 2-heptyl-1*H*-quinolin-4-one (HHQ) through the complex LasR-3-Oxo-C12-HSL on PqsH. LasR positively regulates *rhlR*, again through the complex LasR-3-oxo-C12-HSL and *rhII*. Finally, LasR positively regulates HHQ through *PqsE*. **Elastase** and **protease** exert their effect on disruption of the epithelial barrier and matrix protein (collagen, elastin, etc.). **ToxinA** induces cell death favoring the establishment of infection and colonization. The **alkaline protease** is involved in degradation of the host complement system and cytokines, playing a role in immune evasion and persistent colonization. **Rhamnolipids** favor immune evasion and biofilm formation. **Hydrogen cyanide** reduces lung function. **Pyocyanin**, among various effects, causes oxidative stress and, like **lectinA**, induces paralysis of airway cilia. **RpoS** is a negative transcriptional regulator of rhlI.

*P. aeruginosa* has three main QS systems, named las, rhl, and pqs. Las and rhl modulate the synthesis of AI’s *N*-(3-oxododecanoyl)-l-homoserine lactone (3-oxo-C12-HSL) and *N*-butanoyl-l-homoserine lactone (C4-HSL) as their autoinducers, respectively (Figure 1).

The third QS system in *P. aeruginosa* is *Pseudomonas* quinolone signal (pqs), that is a non-AHL-mediated QS signaling pathway, using alkyl-4-quinolones (AQs), among which 2-heptyl-3-hydroxy-1*H*-quinolin-4-one (PQS) and 2-heptyl-1*H*-quinolin-4-one (HHQ) are signal molecules [22,23]. Even though different kinds of AIs are used by these QS systems, they are interconnected and modulate the activities of each other. As signal molecules bind to receptor LasR, RhlR, or PqsR they subsequently activate the expression of the QS-related genes of *P. aeruginosa*, regulating the production of virulence factors, such as exoenzymes, proteases, elastases, pyocyanine, rhamnolipds, alginate, EPS etc., and other important cellular processes that allow the bacteria to establish an infection in the host tissue [23].

### 2.2. QS Molecular Signaling Network of Gram-Positive Bacteria

**Figure 2:**
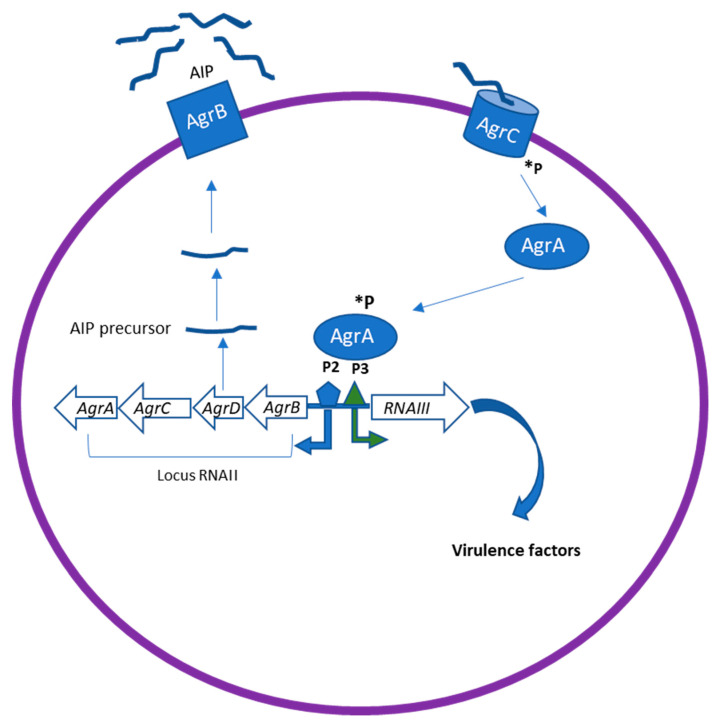
The Agr quorum sensing (QS) system in *Staphylococcus spp*. The Agr locus comprises two divergent transcriptional units, RNAII and RNAIII, containing genes responsible for the production of many virulence factors in *S.aureus*. AgrD encodes the precursor of AIP, which is then processed and transported through AgrB. The processed AIP interacts with a histidine sensor kinase receptor AgrC, which in turn leads to the phosphorylation (*P) of AgrA. This leads to the activation of the regulator AgrA, which binds to the chromosomal P2 and P3 promoter regions to upregulate transcription of RNAII and RNAIII. RNAIII can induce upregulation of virulence factor expression as proteases, toxins, and degradative enzymes.

The Agr system has been identified as the most classical QS system in gram-positive bacteria. It therefore plays a major role in staphylococcal pathogenesis [24]. The Agr locus comprises two divergent transcriptional units, RNAII and RNAIII, containing genes responsible for the production of many virulence factors in *Staphylococcus spp* (Figure 2).

RNAII encodes the core QS circuit protein AgrABCD, whereas RNAIII regulates the expression of multiple virulence genes. *AgrB* and D are involved in the production of the auto-inducing octa-peptides (AIPs) [24]. AgrD encodes the precursor of AIP, which is then processed and transported to the extracellular environment by the integral membrane protease AgrB. When it is released in the environment at high concentration, AIP binds to the kinase receptors (AgrC) on the bacteria membrane, which in turn leads to the phosphorylation of AgrA. This leads to the activation of the regulator AgrA, which binds to the chromosomal P2 and P3 promoter regions to upregulate transcription of RNAII and RNAIII [24]. RNAIII is thus the intracellular effector of the Agr system. Agr can downregulate the expression of cell surface-associated proteins (microbial surface components recognizing adhesive matrix molecules, MSCRAMMs) and upregulate the expression of virulence factors, including toxins (phenol-soluble modulins PSMs, alpha-toxin, delta-toxin (hld), etc.) and degradative exoenzymes (proteases SspA, SspB, Spl, etc.). In addition, Agr induces an increased expression of methicillin resistance genes [25]. Inhibition of AgrA and RNAIII transcription represent an effective strategy for suppressing the virulence of *S. aureus*.

## 3. Anti-Biofilm Activity of Natural Compounds Against *Pseudomonas aeruginosa*

**Figure 3:**
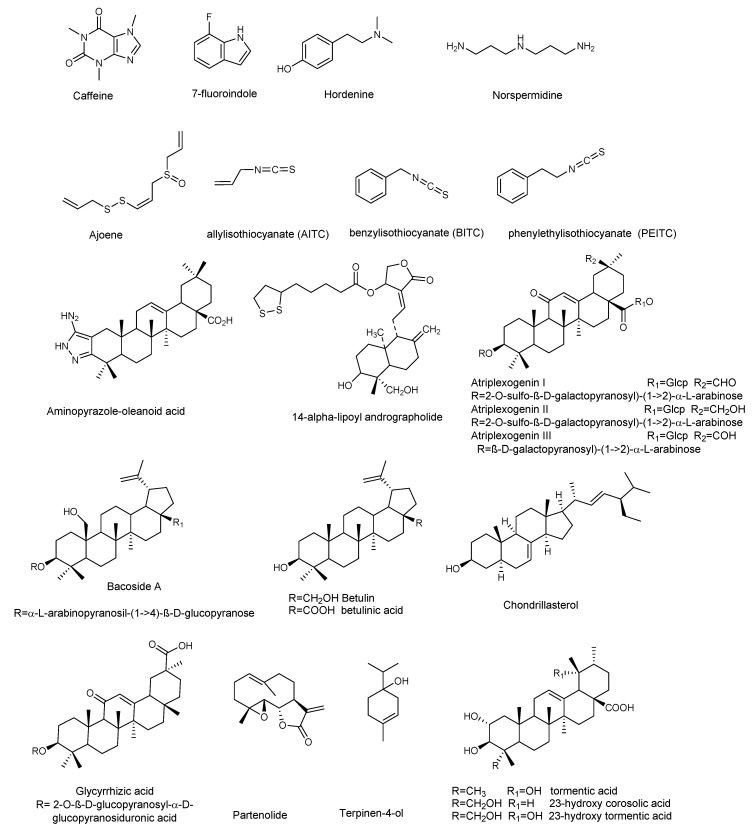
Chemical structures of compounds containing nitrogen, organosulfur compounds, and terpenoids active against *P. aeruginosa*.

**Figure 4:**
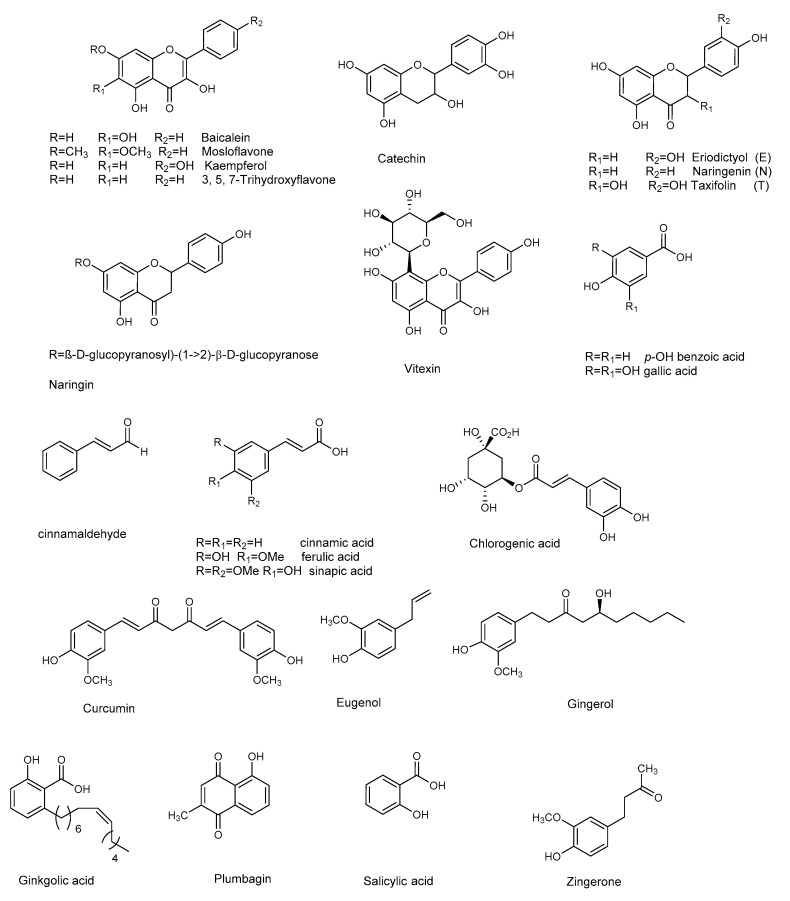
Chemical structures of flavonoids and other phenolic compounds active against *P. aeruginosa*.

*P. aeruginosa* is responsible for a wide range of opportunistic infections, but more complicated to manage are the biofilm-related nosocomial infections, including cystic fibrosis, urinary tract, and eye and burn wounds in immunocompromised patients. Increasing incidents of resistant biofilm infection have resulted in high mortality rates worldwide. Plant-derived anti-biofilm products identified against *P. aeruginosa* include alkaloids, organosulfur compounds, flavonoids, phenolic compounds, and terpenoids (Figure 3 and Figure 4). A number of natural products have been tested for their anti-biofilm potential using mainly crystal violet, or safranin staining method, the evaluation of QS-related antivirulent activity, as well as the capacity to eradicate preformed-biofilm. The section below describes the molecular mechanisms associated with the anti-biofilm effects of the above-mentioned classes of natural products.

### 3.1. Alkaloids and Nitrogen-Containing Compounds

Alkaloids, a large group of basic (mostly) heterocyclic nitrogen containing natural products, are promising candidates for drug discovery. Hordenin is a dietary phytochemical from sprouting barley, traditionally known for its properties as an antimicrobial compound, inhibitor of monoamine oxidase B, stimulator of gastrin release, and as a vasoconstrictive [26]. Recently, Zhou et al. investigated the properties of hordenine as a QS-inhibitor anti-biofilm agent and as an aminoglycoside antibiotic-accelerant against *P. aeruginosa* PAO1 [27]. Hordenine reduced AHLs production and, subsequently, biofilm formation, motility, and virulence factors as protease, elastase, rhamnolipid, pyocyanin, and pyoverdine (Table 1 and Table 2), all important indicators of QS operon in *P. aeruginosa*. The authors analyzed the effect of hordenine on the expression of four QS-related genes, that is *lasI*, *lasR*, *rhlI*, and *rhlR*, in *P. aeruginosa* PAO1. They observed a significant down-regulation of all genes after exposure to 1.0 mg/mL of hordenine. The interest of these results lies in the ability of hordenine to work as a competitive inhibitor of QS (Table 2), exerting a fine gene regulation of major virulence factors in *P. aeruginosa*, thus contrasting the infection. Rhamnolipids are a class of glycolipids regulated by the rhl system (Figure 1), and play a vital role in surface motility and biofilm initiation. They are important bacterial surfactants and a key virulence determinant in *P. aeruginosa* [28]. Rhamnolipids facilitate the degradation of the biofilm matrix and activate the motility to favor the metastatic colonization of new sites. Moreover, production of rhamnolipids by *P. aeruginosa* that colonizes intubated patients was associated with the development of ventilator-associated pneumonia [29]. Other alkaloids were reported for their antibiofilm effects: caffeine [30] and 7-fluoro indole, a synthetic indole-derivative [31]. Both compounds significantly inhibited the biofilm development of *P. aeruginosa* and interfered with the QS by targeting swarming, motility, and several virulence factors.

Polyamines are small organic nitrogen-containing compounds, positively charged at the physiological pH required for normal cell growth in both eukaryotes and prokaryotes [32]. Among them, norspermidine displayed remarkable properties, since it can inhibit the formation of *P. aeruginosa* biofilm and eradicate established biofilms [33]. Norspermidine significantly inhibited the transcription level of lasR/I, rhlR/I, and mvfR, and modulated the QS-related virulence factors (pyocyanin, elastase activity, and protease) [33].

### 3.2. Terpenoids

Terpenes are a wide group of natural compounds characterized by enormous structural diversity, and originating from the coupling of isoprene units. Monoterpenes present in essential oils as well as di- and triperpenoids have long been used as natural medicaments, because of their antimicrobial and anti-biofilm properties. Terpinen-4-ol, the main bioactive constituent of tea tree oil, exhibited QS inhibition at sub-MIC (sub minimum inhibitory concentration) values [36], and reduced the expression of QS genes (*lasI*, *lasR*, *rhlI*, *rhlR*, *rhlAB*, *lasB*, *aprA*, *toxA*, and *plcH*). Further analyses showed the decrease of virulence factors in treated *P. aeruginosa* PAO1, thus confirming the results of QS gene expression analyses. Moreover, Terpinen-4-ol acted synergistically when used in combination with ciprofloxacin, enhancing the effectiveness of the antibiotic against *P. aeruginosa*. This feature makes this natural product very remarkable, because the combined use of old drugs in association with a new antimicrobial, able to potentiate or restore their efficacy, appears as a good strategy to safeguard the future effectiveness of critically important antibiotics.

Parthenolide (Figure 3) is a sesquiterpene lactone obtained from *Tanacetum parthenium*, a plant with well-known medicinal properties, attributable to the active components, sesquiterpenes and sesquiterpene lactones [37]. A study by Kalia et al. [38] demonstrated the ability of parthenolide to contrast *P. aeruginosa* PAO1 biofilm formation, reducing the production of 3-oxo-C12 HSL. Significant decrease in virulence factors and biofilm formation was observed when *P. aeruginosa* was treated with a sub-MIC concentration (Table 4) of parthenolide. At this concentration bacterial growth was not affected. Real time PCR demonstrated the down-regulation of autoinducer synthases (lasI, rhlI), as well as their receptors (LasR and RhlR), correlated with the down-regulation of various virulence factors like pyocyanin, protease, and swarming (Table 3 and Table 4). All the analyzed virulence factors were reduced to a level equivalent to that of the double negative mutant ΔlasIΔrhlI. The addition of autoinducers restored the virulence phenotypes, thus suggesting that parthenolide might interfere with either the synthesis or the reception of AHL. Finally, molecular docking studies evidenced the binding of parthenolide to the active site of the LasR, which may be responsible for the repression of its expression.

Plant derived triterpenes also represent a good scaffold for the synthesis of analogues with improved activities. Indeed, a series of analogs of oleanolic acid showed efficacy in inhibiting biofilm formation and swarming against clinical isolates of *P. aeruginosa* [39]. In particular, the aminopyrazole analog (Table 3) demonstrated potent anti-swarming activity against different strains of gram-negative clinical and agricultural isolates. Notably, the authors analyzed the levels of 24 motility genes in *P. aeruginosa* HONKR grown on swarm plates, with or without this aminopyrazole analog, demonstrating a dose- and time-dependent reduction of gene expression of *algR*, whose product regulates *P. aeruginosa* virulence factors, including type IV pili. The results suggest that the aminopyrazole analog of oleanolic acid acts by interfering with regulation of genes for Type IV pili, bacterial appendages required for motility, thus representing a good candidate for the treatment of persistent *P. aeruginosa* lung infections in cystic fibrosis patients.

Ghosh et al. [40] investigated the anti-biofilm properties against *P. aeruginosa* of tormentic acid and 23-hydroxycorosolic acid, two ursane triterpenes isolated from *Sarcochlamys pulcherrima* (Roxb.) Gaud, an ethnomedicinal plant traditionally used for its anti-microbial and anti-inflammatory properties [48]. Ghosh et al. [40] observed that tormentic and 23-hydroxycorosolic acids (Figure 3) inhibited the growth of planktonic *P. aeruginosa* MTCC 2488 bacteria at MIC of 55 and 40 μg/mL, respectively, in comparison to untreated control. At sub-inhibitory doses, they did not inhibit bacterial growth, while being effective at reducing biofilm formation. Both compounds significantly increased the membrane potential of *P. aeruginosa* at the MIC values, enhancing cell membrane damage and, consequently, cell death. Notably, tormentic and 23-hydroxycorosolic acids reduced the swarming motility and the secretion of proteases and pyoverdine (Table 3 and Table 4), and in vitro and in vivo toxicity studies suggested that they were non-toxic. It was also observed that the treatment with these two triterpenes significantly reduced the bacterial load on a catheter, as well as in liver and spleen. The authors demonstrated that both triterpenoids reduced *lasR*, *lasI*, *lasB*, *rhlI*, and *rhlR* gene expression with respect to the untreated control. These genes are all interconnected and represent a valid tool to verify the QS modulation by natural compounds. The *lasB* gene encodes the metalloproteinase elastase, an important virulence factor in *P. aeruginosa*, since a *lasB* mutation decreases the virulence of the bacterium [49]. It is under the transcriptional control of *lasI*, which encodes a synthase that leads to formation of 3*O*–C12-HSL. The latter diffuses toward the surrounding cells initiating QS, interacts *lasR* with the transcription factor, and activates multiple virulence genes, including *lasB*. In silico docking studies with proteins, like the las family (*lasA*, *lasI*, and *lasR*), *luxR*, and *pil* family (*pilB*, *pilT*, and *pilY1*), showed that tormentic and 23-hydroxycorosolic acids [40], as well as a third ursane triperpene, 23-hydroxytormentic acid from *Mussaenda roxburghii* [41], have good binding affinity with all the selected proteins.

Other pentacyclic triterpenes [42], such as aglycones or saponins [43,44,45], significantly inhibited the formation of *P. aeruginosa* biofilm. Atriplexogenin I-III, oleanane-type saponins from *Atriplex tatarica* [44] in combination with ampicillin and streptomycin acted synergistically, enhancing the effectiveness of antibiotics against *P. aeruginosa*. The same effect was shown by glycyrrhizic acid in combination with ciprofloxacin [45].

### 3.3. Organosulfur Compounds

Jakobsen et al. [35] reported the anti-biofilm properties of ajone, an organosulfide which represents a natural remedy for some human diseases. To determine the QSI (QS Inhibitor) activity of ajoene the authors performed fine experiments by using three reporter systems, which contain fusions of the QS-controlled *lasB* promoter and *rhlA* promoter to *gfp* (ASV), encoding an unstable GFP variant in a *P. aeruginosa* background. The third was a QS reporter system harbored in an *E. coli* background, where the *luxR* gene and the promoter region of the *luxI* were fused to *gfp* (ASV). Microarray analysis showed that ajoene induced a concentration-dependent down-regulation of a few, but central, QS-controlled virulence genes of *P. aeruginosa* (*lasA*, *chiC*, *lecA*, *rhlA*, *rhlB*, *prpL*, *cbpD*), with the best activity at 80 μg/mL. Attempts to repress more genes were successful only with higher concentrations, also affecting cell growth. DNA microarray studies represent an important tool in the investigation of a plethora of QS-regulating genes. Microarray data were confirmed by RT-PCR analysis of two QS-regulated genes *lasB* and *rhlA*. Due to *rhlA* gene down-regulation, the rhamnolipid content was drastically reduced when the cells were treated with 80 μg/mL ajoene (Table 2). Ajoene demonstrated a clear synergistic effect, with tobramycin killing bacteria embedded in biofilm, and inhibited the lytic necrosis of polymorphonuclear leukocytes. Furthermore, during in vivo studies on a mouse model of pulmonary infection, a significant clearing of infecting *P. aeruginosa* was detected in ajoene-treated mice compared to a nontreated control group.

Isothiocyanates, another class of compounds containing sulfur, known for their antimicrobial activity, also showed significant activity in the treatment of biofilm-related infections caused by *P. aeruginosa*. In particular allylisothiocyanate (AITC), benzylisothiocyanate (BITC), and phenylethylisothiocyanate (PEITC), found in plants such as nasturtium (*Tropaeolum majus*) and horseradish (*Armoracia rusticana*), were analyzed on mature and developing biofilms of clinical *P. aeruginosa* (blood culture isolates, multidrug-resistant (MDR) and extensively drug-resistant (XD) Pa strains from invasive and non-invasive clinical samples) isolated either from clinical patients with signs and symptoms of infection, or from the hospital environment [34]. PEITC was the most effective on the development of *P. aeruginosa* biofilms (500 µg/mL) while AITC preparations showed effectiveness on established *P. aeruginosa* biofilms, reducing their metabolic activity (between 200 and 800 μg/mL) to a level comparable to the mixture of all three compounds (ITCM, 500–1000 μg/mL). The combination of isothiocyanates with the antibiotic meropenem showed a synergistic effect, with better results when compared to either preparation alone [34].

### 3.4. Flavonoids

Flavonoids are natural products ubiquitously present in the plant kingdom. They are classified based on the chemical functionalization of the C ring in: flavones (α-β unsaturated ketone), flavanones (ketone at C-4), flavonols (the 3-hydroxy derivative of flavones), and flavan-3-ol (hydroxyl at C-3). These compounds are often also present in glycoside form. Several flavonoids have been evaluated for their anti-biofilm activities, mainly QS-activities. Baicalein (Figure 4) is the most abundant flavone monomer extracted from the roots of *Scutellaria baicalensis*, and used as a medicine in the Chinese Pharmacopoeia for the treatment of fever, sore throat, and upper respiratory tract infection [50,51]. Baicalein is commercially produced as oral tablets for the treatment of bacteria-induced diarrhea. In addition to its antimicrobial properties baicalein has demonstrated important anti-inflammatory properties [52]. The latter is an important result since a hallmark of *P. aeruginosa* pulmonary infection is the secretion of various proinflammatory cytokines and a massive recruitment of neutrophils to the infection site. Such excessive inflammatory responses are harmful to the host, contributing to severe tissue damage and organ dysfunction. Therefore, the contemporary administration of an anti-inflammatory drugs is necessary, to slow the progression of chronic infectious diseases by interrupting the infection and inflammation.

Along with anti-QS activity (attenuation of *P. aeruginosa* virulence factors, including swarming and twitching, and down-regulation of QS-regulated genes transcription) baicalein (128 μg/mL) significantly attenuated IL-1β, IL-6, IL-8, and TNFα secretion at sub-MIC level compared with the PAO1-infected group in the absence of baicalein treatment. At the same concentration, baicalein significantly prevented *P. aeruginosa*-induced IκBα phosphorylation and the subsequent nuclear translocation and DNA-binding activity of NFκB (p65), compared to the untreated *P. aeruginosa* PAO1. In summary, the results showed that baicalein represents a promising candidate for combating *P. aeruginosa* infection, since it can attenuate bacterial pathogenesis by interfering with the QS system, and for its notable anti-inflammatory effect. The flavanones naringenin and taxifolin [53], as well as the flavan-3-ol catechin [54], also showed promising anti-biofilm properties, due to the ability to reduce the production of QS-controlled virulence factors in *P. aeruginosa* PAO1 (e.g., pyocyanin and elastase) and to modulate the expression of several QS-controlled genes (Table 5 and Table 6). Naringin, a glycoside of naringenin, was screened for its capacity to inhibit the QS-controlled factors, and its antibiofilm efficacy by Vandeputte [53], and recently by Dey et al. [55]. Although naringin showed antibiofilm activities, in addition to its combinatorial performances with antibiotics ciprofloxacin and tetracycline [55], RT-PCR showed that this compound did not reduce the expression of any of the selected QS genes (*lasI*, *lasR*, *lasB*, *rhlI*, *rhlR*, *rhlA*, and *aceA*) [53].

Thanks to the low risk and contextual multitargeted actions, the combination of nanoparticles (NPs) and natural compounds has gained a lot of attention in biomedical applications [58]. Zinc and copper play vital roles in several biological processes. Due to their biomedical applications and selective binding to phytochemicals, zinc oxide nanoparticles or zinc and copper thin film techniques are becoming attractive in biomedical applications. Recently, flavonoid-loaded nanoparticles were assessed for their anti-biofilm properties in order to evaluate the potential antibacterial effects, in comparison to the parent flavonoid. In particular, the use of dual drug-like molecules (rutin-benzamide) loaded in a poly vinyl alcohol (PVA) surface modified single nanocarrier (PEG−PLGA) represents a potential anti-biofilm therapy, based on interesting results in term of EPS reduction as well as the extent (%) of biofilm inhibition compared to the control [59]. In addition to pure flavonoids, crude extracts containing flavonoid derivatives as principal constituents also attenuated QS-mediated virulence and biofilm formation. In particular, the binding affinity of mosloflavone for RhlR, detected in the methanolic extract of *Plectranthus tenuiflorus* [60], was observed to be comparatively higher than its natural ligand, while kaempferol constituted the major constituent of *Centella asiatica*, a herb with proven anti-QS properties [61].

### 3.5. Other Phenolic Compounds

Curcumin, present in the rhizome of turmeric (*Curcuma longa* L.), has many properties and a long-term use in traditional Indian medicine as an antimicrobial agent [62]. Anti-biofilm properties (see Table 7) at sub-MIC concentration are ascribed to curcumin, that down-regulate the *P. aeruginosa* PAO1 QS system and related virulence factor (pyocyanin, protease and elastase, Table 8) [63]. To overcome its poor water solubility, and enhance its antimicrobial properties, curcumin has been loaded onto zinc oxide nanoparticles (ZnO-NCs), excellent drug carriers due to their low toxicity and biodegradable nature [64]. This considerably improved the anti-QS effect of curcumin against *P. aeruginosa* PAO1. ZnC-NCs suppressed the LasR-RhlR transcriptional activators and was capable of triggering ROS generation. The ZnC-NC-induced O^2−^ generation was responsible for its anti-biofilm effect against *P. aeruginosa* PAO1. Molecular docking analysis confirmed the molecular mechanism, showing how curcumin better fits inside the binding site of LasR protein (−5.9730) and RhlR protein (−6.5435).

Another natural product showing good antibacterial and anti-biofilm properties against *P. aeruginosa* (MTCC 424, MTCC 2488) is the naphthoquinone plumbagin [65]. This compound has been used as a traditional medicine in India for its antiparasitic, antioxidant, anticancer, and antimicrobial properties, and can be isolated from the roots of Plumbaginaceae plants [66]. It was demonstrated that plumbagin alone, and in combination with gentamicin, significantly reduced the secretion of virulent enzymes and virulence factors against both strains of *P. aeruginosa*. The expression of *lasB*, *lasI*, and *lasR* genes was also significantly reduced following plumbagin treatment of *P. aeruginosa* MTCC 424 and MTCC 2488, at 250 and 150 μg/mL, respectively. In addition, plumbagin showed a synergistic interaction with gentamicin. This combinatorial approach, which represents a novel strategy for the reduction of biofilm formation by *P. aeruginosa,* also encourages the use of existing antibiotics at lower doses. Plumbagin’s mechanism of action was assessed by protein-ligand docking analysis. The compound showed good affinity for the ligand binding site of Las family and Pil family proteins: the former is related to QS, while the latter to pilus assembly. This result led the authors to hypothesize that plumbagin may affect pilus assembly, inhibiting the QS and swarming motility.

Many other phenol derivatives (Figure 4) exhibited remarkable anti-biofilm properties (Table 7 and Table 8) against *P. aeruginosa*: cinnammic acid [67], ginkgolic acid [68], gallic, chlorogenic, sinapic, and ferulic acids, as well as eugenol [69,70,71]. Moreover, synergistic effects due to the combination of two or more phenolic compounds have been detected, as in the case of salicylic acid and trans-cinnamaldehyde [72].

## 4. Anti-Biofilm Properties of Natural Compounds against *Staphylococcus aureus*

**Figure 5:**
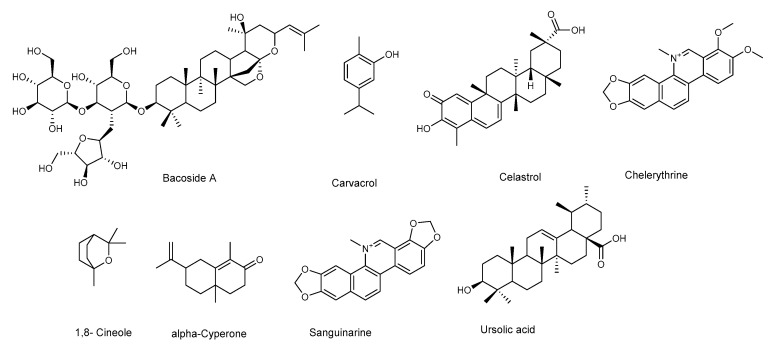
Chemical structures of terpenoids active against *Staphylococcus aureus*.

**Figure 6:**
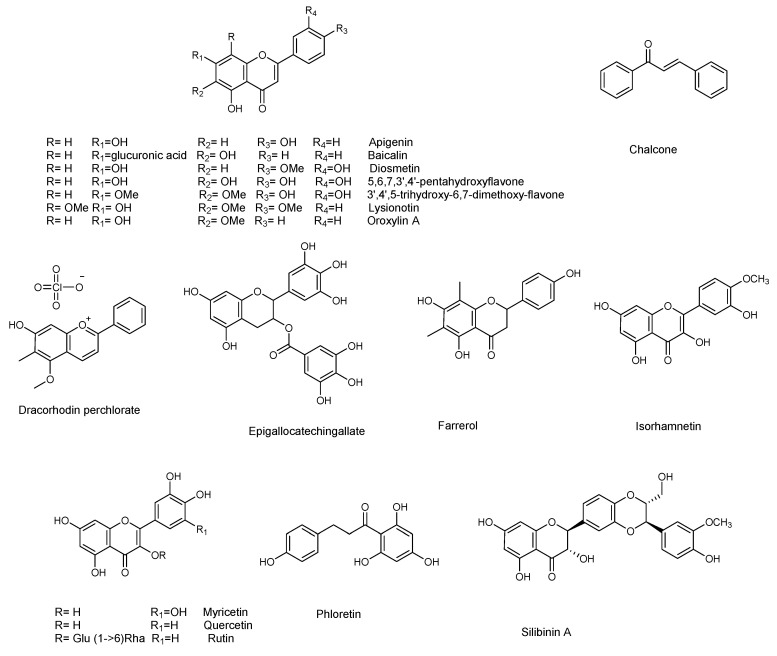
Chemical structures of flavonoids active against *S. aureus*.

*Staphylococcus aureus* is a gram-positive pathogen, frequently the cause of biofilm-associated infections on indwelling medical devices [77]. Like for *P. aeruginosa*, staphylococcal biofilms show enhanced resistance toward antibiotics and the immune response, thus representing an important therapeutic challenge in clinics worldwide. A recent study has already provided an accurate overview of natural products isolated from plants and microorganisms with activity against the major virulence factors of *S. aureus* [78]. In this section, we report the latest updates and, when the information is available, the molecular mechanisms associated with the anti-biofilm effects of terpenes, flavonoids, and phenolic compounds (Figure 5 and Figure 6, Table 9, Table 10 and Table 11).

### 4.1. Terpenes

Among monoterpenes, 1,8-cineole (Figure 5) and carvacrol [79,80] were shown to act against biofilm formation, while eugenyl acetate was active against alfa-hemolysin [81].

The sesquiterpene (+)-nootkatone is present in essential oils from Alaska yellow cedar trees, some herbs, and grapefruit. It has been approved by the Food and Drug Administration (FDA) as a flavoring agent in citrus-flavored foods and beverages. Farha et al. [82] demonstrated that (+)-nootkatone at 200 μg/mL significantly disrupted *S. aureus* preformed biofilm, and reduced the viability of cells within matured biofilm, suggesting that the compound penetrates through the biofilm. Additionally, the molecular analysis showed that (+)-nootkatone suppressed the expression levels of *sarA*, *icaA*, *agrA*, *RNAIII*, and *spa*; major genes involved in biofilm formation. The compound was also able to inhibit the sliding motility of *S.aureus*, thus contrasting the initial phase of bacterial surface colonization and biofilm formation. Moreover, up to 50 μg/mL, sub-MIC concentration, at which the inhibition of biofilm formation was observed, (+)-nootkatone was non-toxic to normal fibroblast cells.

The diterpenes, salvipisone and aethiopinone, isolated from hairy roots of *Salvia sclarea*, showed activity against methicillin-resistant *S. aureus*. They reduced the resistance to the antibiotic oxacillin, and caused a reduction of the biofilm biomass, as well as the disruption of the biofilm structure [83].

The triterpene celestrol (Table 9) was shown to inhibit biofilm formation, and to possess antimicrobial activity against *S. aureus* ATCC 29,213 (a reference strain of methicillin-sensitive *S. aureus* (MSSA)) and a clinical methicillin-resistant *S. aureus* (MRSA) isolate [84]. The compound was not only active on planktonic cells (with a MIC of 2 µM and a MBC of 32 µM), but it was also effective in dispersing preformed biofilms of the clinical MRSA isolates, as evaluated by confocal laser scanning microscopy. Furthermore, it inhibited the secretion of EPS, which are crucial for the formation of the matrix that facilitates the adherence of these microorganisms on the target surfaces. Therefore, the compound has a great potential, since it is not only inhibiting to the formation of biofilms, but it can further act by eradicating preformed biofilms, while also being active on the planktonic cells. However, this compound also showed a certain cytotoxicity against hFOB 1.19 cells (osteoblast).

Ursolic acid (Table 9) was active against the formation of biofilm by *S. aureus* subsp. *aureus* COL, a MRSA strain, resistant to several antibiotics, including penicillin and tetracycline. The RNA-Seq-based transcriptome analysis showed that ursolic acid reduces the metabolism of some amino acids and the expression of adhesins [85].

A mixture of triterpenoid saponins, known as Bacoside A (Figure 5), was reported for its antimicrobial and anti-biofilm activity against *S. aureus* MTCC 96. It is very likely that these saponins alter the structure and permeability of the bacterial cell membrane. Furthermore, Bacoside A, also dispersed preformed biofilm. The treated biofilm showed altered cell structure and a loss of EPS that caused biofilm dispersion [43].

### 4.2. Flavonoids

Among the flavonoids, baicalein (which is also active against *P. aeruginosa*) was active against the QS system, by inhibiting the transcription of AgrA and RNAIII, and inhibits biofilm formation [88]. The biofilm formation was also inhibited by myricetin [89]. Myricetin, quercetin, farrerol, isorhamnetin, dracorhodin, lysionotin, diosmetin, silibinin, apigenin, epicallocatechin gallate, oroxylin A, and baicalin (Figure 6) were active against alfa-haemolysin [89,90,91,92,93,94,95,96,97,98,99,100]. The flavonoid rutin showed a concentration dependent reduction of biofilm formation (Table 10). However, it did not significantly decrease the biomass, while it reduced the secretion of EPS. Therefore, it probably acts by interfering with the adhesion, and with all the other functions, of EPS [101]. Pro-antocyanidin A2 inhibited de-novo biofilm formation, without showing bactericidal activity, nor inhibiting activity on planktonic growth. Furthermore, it also appeared to have no activity on mature biofilm [86]. Two flavonoids isolated from *Teucrium polium*, namely 3′,4′,5-trihydroxy-6,7-dimethoxyflavone and 5,6,7,3′,4′-pentahydroxyflavone, inhibited biofilm growth of *Staphylococcus aureus* (Table 10) AH133 strain [102]

A recent study explored the capacity of kaempferol to inhibit *S. aureus* biofilm formation, and the associated potential molecular mechanisms [103]. Kaempferol inhibited the attachment phase of biofilm formation, by reducing *S. aureus* adhesion, since its action was evident only if added immediately after the inoculation of bacteria to plates. This was mediated by blocking the activity of Sortase A (SrtA), an enzyme essential in the anchoring of surface proteins to the cell wall of gram-positive bacteria. This has important consequences for the onset of acute infection by *S. aureus*, since the bacteria cannot display functional surface adhesins in the cell wall envelope. In addition, the authors analyzed the expression of adhesion-related genes. They demonstrated that the compound reduced the expression of *clfA* and *clfB*, which encode clumping factor A (ClfA), and ClfB, *fnbA*, and *fnbB* which encode fibronectin-binding proteins (FnbpA and FnbpB), and *sarA*, a global regulator gene that is closely related to biofilm formation, and positively regulates *fnbA* and *fnbB*. The results reported suggest that kaempferol represents a potential compound with a novel mechanism of biofilm inhibition.

### 4.3. Other Phenolic Compounds

Several other plant-derived phenolic compounds have already been discussed in the work by Wu et al., including chalcone [106], resveratrol ([107], phloretin [104], alfa-cyperone [87], curcumin [108], osthole [105], and brazilin [109]. Among these, resveratrol (Table 11) was active against α-hemolysin [107] and, used in combination with vancomycin, inhibited biofilm formation. It was suggested that resveratrol would disturb the expression of genes related to QS, surface and secreted proteins, and capsular polysaccharides [85].

Besides these, gallic and ferulic acid were also tested for their activity against *S. aureus*, although they were active only at relatively high concentrations. Only ferulic acid completely inhibited colony spreading. Furthermore, it was hypothesized that changes in motility could affect the ability of the bacteria to form a biofilm [71]

The tannin, hamamelitannin, was shown to inhibit the quorum sensing regulator RNAIII [110,111], while punicalagin was active against α-hemolysin [112], and exerted a remarkable inhibitory effect on biofilm formation [113]. The activity of punicalagin against *S. aureus* was further investigated, with the aim of understanding the possible mode of action. Punicalagin exhibited a MIC of 0.25 mg/mL and induced morphological damage to the cell membrane, also inducing an efflux of potassium.

Tannic acid showed antibacterial and anti-biofilm formation activity, although further studies are needed to understand the mechanism of action [114].

1,2,3,4,6-Penta-*O*-galloyl-d-glucopyranose (PGG) prevented biofilm formation at 6.25 µM of several strains of *S. aureus*, while showing no bactericidal activity at this concentration [115]. Arylbenzylfuran was active against clinical strains of methicillin-resistant S. aureus (MRSA), and was able to induce a significant reduction in S. aureus ATCC 12600S biofilm viability [116].

Several aromatic polyketides isolated from plants have been reported, in particular aloe-emodin [117], acting against the Agr quorum-sensing system. The same compound and the structurally related rhein [117] were able to inhibit biofilm formation.

Finally, noteworthy is the activity of capsaicin, which acts against α-hemolysin by suppressing the expression of *Hla* and *AgrA* [95].

## 5. Conclusions

Biofilms represent one of the most successful strategies used by bacteria to increase their survival in terms of resistance to antibiotics and antimicrobial agents. If biofilm forming microorganisms are a big challenge, even more complex is the fight against polymicrobial biofilms, like the ones formed by *S. aureus* and *P. aeruginosa*. Therefore, finding new anti-biofilm chemicals is crucial. In this context, plants are an extraordinarily rich source of compounds endowed with several different biological activities, including antimicrobial and antibiofilm properties. These compounds often act via modes of action that are different than the ones of currently used antibiotics, thus also offering a tool for combating antibiotic resistance.

Many studies have been published on the topic in recent years, and the latest advances in the discovery of plant-derived natural products with anti-biofilm properties against *P. aeruginosa* and *S. aureus* have been herewith reviewed. Knowing the molecular mechanisms underlying the biological activity is very important, especially if these compounds are to be further studied for possible applications. Therefore, when known, the molecular mechanisms were also herewith reported and discussed, with the aim of providing a clear overview of the state of the art.

## Figures and Tables

**Table 1:** Compounds containing nitrogen and sulfur inhibiting biofilm formation and motility in *P. aeruginosa* ^1^.

Compounds	BIOFILM ASSAY					MOTILITY ASSAY			REF.
	Total Protein Content	Crystal Violet (CV) or Safranin (S) Staining	Metabolic Activity of Biofilm	EPS Production	Pre-Formed Biofilms	Swarming	Swimming	Twitching	
Hordenine		↓31% (1 mg/mL)			↓23% (1 mg/mL)	↓54% (0.5–1.0 mg/mL)	↓82% (0.5–1.0 mg/mL)		[27]
7-fluoroindole		↓76% (1 mM)				abolished swarming (1.0 mM)	no effect	no effect	[31]
Caffeine	↓~30% (80 µg/mL)	↓~50% (80 µg/mL)		↓~50% (80 µg/mL)		↓ (40–80 µg/mL)			[30]
Norspermidine		↓48–90% (10 mmol/L)			↓43–85% (10 mmol/L)		↓ 42.4% (4 mmol/L)		[33]
Allylisothio-cynate		no effect (50 μg/mL)	↓65–70% (200–800 μg/mL)						[34]
Benzyl-isothiocyanate		no effect (50 μg/mL)	↓70% (250–1000 μg/mL)						[34]
Phenylethyl-isothiocyanate		↓40% (500 μg/mL)	↓30–60% (60–240 μg/mL)						[34]

^1^ Down arrow (↓) indicates decrease of tested activity with respect to the control (ctr). Tested concentrations are reported in parenthesis. White box shows a not performed assay.

**Table 2:** Compounds containing nitrogen and sulfur inhibiting *P. aeruginosa* virulence factors regulated by QS ^1^.

Compounds	VIRULENCE FACTORS Regulated by QS								REF.
	AHLs Levels	Alginate Assay	Elastase Assay	Hemolysin Assay	Protease Assay	Pyocyanin Assay	Pyoverdine Secretion Assay	Rhamno-Lipid	
Hordenine	↓79% C4-HSL, ↓66% 3-oxo-C12-HSL (1 mg/mL)	↓60%, Res↓ 50%: (1 mg/mL)	↓65%, Res ↓30%: (1 mg/mL)		↓61%, Res ↓40% (1 mg/mL)	↓80%, Res ↓40%: (1 mg/mL)	↓65%, Res ↓40%: (1 mg/mL)	↓53%, Res ↓40%: (1 mg/mL)	[27]
7-fluoroindole				↓93% (1 mM)		↓ marked reduction	↓ marked reduction	↓ marked reduction	[31]
Caffeine					↓70% (80 µg/mL)	↓~60% (80 µg/mL)			[30]
Norspermidine			↓59–69% (0, 2, and 4 mmol/L)		↓53–66% (0, 2, and 4 mmol/L)	↓45–54% (0, 2, and 4 mmol/L)			[33]
Ajoene	↓ 3-fold C4-HSL (80 µg/mL)							↓3-fold (20 µg/mL)	[35]

^1^ Down arrow (↓) indicates decrease of tested activity with respect to the control (ctr). Tested concentrations are reported in parenthesis. White box shows a not performed assay. 3-oxo-C12-HSL AIs *N*-(3- oxododecanoyl)-l-homoserine lactone (3-oxo-C12-HSL) and *N*-butanoyl-l-homoserine lactone (C4-HSL). Res (resveratrol, positive control).

**Table 3:** Terpenoids inhibiting biofilm formation and motility in *P. aeruginosa* ^1^.

Compounds	BIOFILM ASSAY				MOTILITY ASSAY			REF.
	Total Protein Content	Crystal Violet (CV) or Safranin (S) Staining	EPS Production	Pre-Formed Biofilms	Swarming	Swimming	Twitching	
Terpinen-4-ol		↓ (0.06% *v*/*v*)		↓ young, peak mature biofilm	↓33,3% (0.06% *v*/*v*)	↓50% (0.06% *v*/*v*)	↓25% (0.06% *v*/*v*)	[36]
Parthenolide		↓56% (1 mM)			↓(1 mM)			[38]
Aminopyrazole- oleanoid acid					↓>85% (1 µg/mL)			[39]
Tormentic acid	↓25.4% (25 µg/mL)	(S)↓53.8% (25 µg/mL)	↓39.2% (25 µg/mL)		↓6.8 mm (25 µg/mL)			[40]
23-OH corosolic acid	↓28.7% (20 µg/mL)	↓55.6% (20 µg/mL)	↓41.9% (20 µg/mL)		↓6.2 mm (20 µg/mL)			[40]
23-OH tormentic acid	↓37.1% (0.37 mM)	(S) ↓37.6% (0.37 mM)	↓36.9% (0.37 mM)		↓2 ± 0.4 (0.21 mM)			[41]
Betulin		↓57.3% (125 µg/mL)	↓31.3% (125 µg/mL)		↓47.3% (125 µg/mL)	↓ (125 µg/mL)	↓ (125 µg/mL)	[42]
Betulinic acid		↓33.0% (125 µg/mL)	↓35.2% (125 µg/mL)		↓ 51.4% (125 µg/mL)	↓ (125 µg/mL)	↓ (125 µg/mL)	[42]
Bacoside		↓90% (200 µg/mL)		20% cell viability (200 µg/mL)				[43]
Atriplexogenin I		↓7.2–56.2% (125–500 µM)						[44]
Atriplexogenin II		↓12.5–26.5% (62.5–250 µM)						[44]
Atriplexogenin III		↓39.4–53.4% (345–690 µM)						[44]
Glycyrrhizic acid		↓65.1–83.3% (50–200 mg/mL)						[45]
Chondrillasterol		↓>90% (100 µg/mL)		↓>60% (1.6–100 µg/mL)				[46]
14-alpha-lipoyl andrographolide		↓ (0.5 mM)	↓ (0.5 mM)					[47]

^1^ Down arrow (↓) indicates decrease of tested activity with respect to the control (ctr). Tested concentrations are reported in parenthesis. White box shows a not performed assay.

**Table 4:** Terpenoids inhibiting *P. aeruginosa* virulence factors regulated by QS ^1^.

Compounds	VIRULENCE FACTORS Regulated by QS							REF.
	Alginate Assay	Elastase Assay	Hemolysin Assay	Protease Assay	Pyocyanin Assay	Pyoverdine Secretion Assay	Rhamno-Lipid	
Terpinen-4-ol	↓56% (0.06% *v*/*v*)	↓50% (0.06% *v*/*v*)	↓60% (0.06% *v*/*v*)	↓5% (0.06% *v*/*v*)	↓33% (0.06% *v*/*v*)			[36]
Parthenolide				↓45% (1 mM)	↓35.1% (1 mM)			[38]
Tormentic acid				↓ 53.0% (25 µg/mL)		↓(20 µg/mL)		[40]
23-OH corosolic acid				↓46.6% (20 µg/mL)		↓(20 µg/mL)		[40]
23-OH tormentic acid				↓37.8% (0.37 mM)				[41]
Betulin	↓88.3% (125 µg/mL)				↓74.5% (125 µg/mL)		↓19.0% (125 µg/mL)	[42]
Betulinic acid	↓ 54.7% (125 µg/mL)				↓54.7% (125 µg/mL)		↓21.6% (125 µg/mL)	[42]
14-alpha-lipoyl andrographolide					↓ (0.5 mM)			[47]

^1^ ↓ indicates decrease of tested activity with respect to the control. Tested concentrations are reported in parenthesis. White box shows a not performed assay.

**Table 5:** Flavonoids inhibiting biofilm formation and motility in *P. aeruginosa* ^1^.

Compounds	BIOFILM ASSAY				MOTILITY ASSAY			REF.
	Total Protein Content	Crystal Violet (CV) or Safranin (S) Staining	Metabolic Activity of Biofilm	EPS Production	Swarming	Swimming	Twitching	
Baicalein		↓35.7% and ↓53% (1 and 5 days) at 128 µg/mL			↓ (128 µg /mL)	no effect	↓ (128 µg /mL)	[52]
Catechin		↓ 30% (4mM)						[54]
Naringin		↓49.5% (410 µg/mL), *Cpr, Tet	↓49.5% (410 µg/mL), *Cpr, Tet	↓ 40% (410 µg/mL), *Cpr, Tet	↓ 42% (410 µg/mL)	↓14% (410 µg/mL)		[55]
3, 5, 7-Trihydroxyflavone			↓76% (100)	↓74,5% (100 µg/mL)		↓(25–100 µg /mL)		[56]
Vitexin		(S)↓56% (110 µg/mL)		↓40% (110 μg/mL)	↓100 µg /mL			[57]

^1^ Down arrow (↓) indicates decrease of tested activity with respect to the control (ctr). Tested concentrations are reported in parenthesis. White box shows a not performed assay. * Synergistic effect with ciprofloxacin (Cpr) and tetracycline (Tet).

**Table 6:** Flavonoids inhibiting *P. aeruginosa* virulence factors regulated by QS ^1.^

Compounds	VIRULENCE FACTORS Regulated by QS							REF.
	AHLs Levels	Crystal Violet (CV) or Safranin (S) Staining	Elastase Assay	Protease Assay	Pyocyanin Assay	Pyoverdine Secretion Assay	Rhamno-Lipid	
Baicalein	↓	(32–128 µg/mL)	↓94.2%, LasB (128 µg/mL)	↓74.56% LasA (128 µg/mL)	↓69.9% (128 µg/mL)		↓74.1% (128 µg/mL)	[52]
Eriodictyol (E), Naringenin (N), Taxifolin (T)	↓3-oxo-C12-HSL and C4-HSL N (4 mM)		↓46% N, 62% E, 47% T (4 mM)		↓87% N, 73% E, ¯56% T (4 mM)			[53]
Catechin			↓30% (4 mM)		↓(0.125 and 16 mM)			[54]
3, 5, 7-Trihydroxyflavone				↓52% (0.1 µg/mL)	no effect			[56]
Vitexin			LasB ↓37.5% (110 µg/mL)	↓39.04% inhibition of LasA	↓ moderate (100 µg/mL)	↓ moderate (100 µg /mL)		[57]

^1^ Down arrow (↓) indicates decrease of tested activity with respect to the control (ctr). Tested concentrations are reported in parenthesis. White box shows a not performed assay. 3-oxo-C12-HSL AIs *N*-(3-oxododecanoyl)-l-homoserine lactone (3-oxo-C12-HSL) and *N*-butanoyl-l-homoserine lactone (C4-HSL).

**Table 7:** Phenolic compounds inhibiting biofilm formation and motility in *P. aeruginosa* ^1^.

Compounds	BIOFILM ASSAY					MOTILITY ASSAY			REF.
	Total Protein Content	Crystal Violet (CV) or Safranin (S) Staining	Metabolic Activity of Biofilm	EPS Production	Pre-Formed Biofilms	Swarming	Swimming	Twitching	
Plumbagin	↓76% (150 µg/mL)	(S)↓60% (150 µg/mL)		↓52% (150 µg/mL)	↓41% (150 µg/mL)	↓55.5% (250 µg/mL)			[65]
Cinnamic acid		↓50.1 (250 µg/mL)							[67]
Curcumin		↓1.5–3 µg/mL							[63]
Eugenol		↓43% at 400 µM				No inhibition (200 μM)			[69]
Gallic acid		↓2- to 2.5-fold				↓20–50%, (400–800 μg/mL)		↓0–15% (400–800 μg/mL)	[70]
4-OH benzoic acid						↓12–30% (400–800 μg/mL)		↓0–15% (400–800 μg/mL)	[70]
Ferulic (FA) cinnamic (CA) acids		↓2- to 2.5-fold CA, FA							[70]
Chlorogenic acid		↓2- to 2.5-fold							[70]
Gallic acid (GA) Ferulic acid (FA)		↓84% GA ↓ 81% FA	¯100% GA, 0% FA			↓42% GA, FA (1 mg/mL)	↓42% GA, 47% FA (1 mg/mL)	↓42% GA, 8% FA (1 mg/mL)	[71]
Salicylic acid (SA) cinnamaldehyde (CIN)		¯26% CIN ¯54% SA							[72]
6-gingerol		↓19–53% (0.1–100 µM)							[73]
Zingerone		↓			↓	↓51.3% (10 mg/mL)	↓53% (10 mg/mL)	53% (10 mg/mL)	[74]
Zingerone		↓ 50% reduction of biofilm				↓55% (10 µg/mL)	↓68% (10 µg/mL)	↓67% (10 µg/mL)	[75]
Proanthocyanidin monomer A-type		↓40.9% (1 μg/mL)	↓36.9% (10 μg/mL)		↓54.1% at 10 μg/mL	↓100 μg/mL			[76]

^1^ Down arrow (↓) indicates decrease of tested activity with respect to the control (ctr). Tested concentrations are reported in parenthesis. White box shows a not performed assay.

**Table 8:** Phenolic compounds inhibiting *P. aeruginosa* virulence factors regulated by QS ^1^.

Compounds	VIRULENCE FACTORS Regulated by QS								REF.
	AHLs Levels	Alginate Assay	Elastase Assay	Hemolysin Assay	Protease Assay	Pyocyanin Assay	Pyoverdine Secretion Assay	Rhamno-Lipid	
Plumbagin					↓40% (150 µg/mL)	↓>80% (150 µg/mL)	↓ (150 µg/mL)		[65]
Cinnamic acid		↓21.8 (250 µg/mL)	↓49.9 (250 µg/mL)		↓ 80.9 (250 µg/mL)	↓71.4 CA (250 µg/mL)		↓16.5 (250 µg/mL)	[67]
Ginkgolic Acid						↓90%			[68]
Curcumin	↓25% in 3-oxoC12-HSL, ↓>2% C4-HSL (1 µg /mL)		↓2-fold (3–5 µg/mL)		↓2-fold vs ctr (3–5 µg/mL)	↓60–80% (1.5–3 µg/mL)			[63]
Eugenol			↓32 and 46% (200and 400 µM)					↓56% at 50 µM	[69]
Salicylic acid (SA) cinnamaldehyde (CIN)			↓22% CIN, ↓28% SA		↓65% CIN, ↓31% SA	↓32% CIN ¯70% SA		significant ↓	[72]
6-gingerol	↓ (0.1–1 mM)				↓21–43% (1, 10, and 100 mM)	↓36–60% (1, 10, and 100 mM)		↓36–60% (1, 10, and 100 mM)	[73]
Zingerone	↓ C4-HSL, OdDHL,		Marked ↓	Marked ↓	↓Significant	↓ Significant		Marked ↓	[75]
Proanthocyanidin monomer A-type									[76]

^1^ Down arrow (↓) indicates decrease of tested activity with respect to the control (ctr). Tested concentrations are reported in parenthesis. White box shows a not performed assay. 3-oxo-C12-HSL AIs *N*-(3- oxododecanoyl)-l-homoserine lactone (3-oxo-C12-HSL) and *N*-butanoyl-l-homoserine lactone (C4-HSL), N-(3-oxododecanoyl)-l-homoserine lactone (OdDHL).

**Table 9:** Terpenoids inhibiting *S. aureus* biofilm formation and production of virulence factors regulated by QS ^1^.

Compounds	BIOFILM ASSAY				VIRULENCE FACTORS Regulated by QS	Ref.
	Cristal Violet (CV) or Safrin (S) Staining	Metabolic Activity of Biofilm-Forming by MTT Assay	EPS Production	Pre-Formed Biofilms	Hemolysin	
Chelerythrine (CH) Sanguinarine (SA)	↓SA (24.5 μM) ↓CH (15.2 μM)					[86]
Celastrol	↓25.5–85.07% ATCC 29213 ↓27–89.3% MRSA (40 μmol/L)		↓40.8–76.0% ATCC 29213 ↓42.0–51.1% MRSA (1.25 μmol/L)	↓40.5–80.2% ATCC 29213 ↓49.5–82.8% MRSA (40 μmol/L)		[84]
Carvacrol	↓(0.50 to 1 mM)			↓(8 mM)		[80]
1,8-Cineole	↓(0.095 mg/mL)			↓77.46 ± 1.91%–90.81 ± 4.05% (0.048, 0.096, 0.192 mg/mL)		[79]
alpha-Cyperone					↓6.3% ATCC 29213; 4.4% BAA-1717; 12.6% Wood 46; 6.1% 83254 (16 μg/mL).	[87]
Ursolic acid	↓66.3% (30 µg/mL)					[85]
Bacoside A	↓90% (200 µg/mL)	↓10% (200 µg/mL)	↓			[43]

^1^ Down arrow (↓) indicates decrease of tested activity with respect to the control (ctr). White box shows a not performed assay.

**Table 10:** Flavonoids inhibiting *S. aureus.* biofilm formation and production of virulence factors regulated by QS ^1^.

Compounds	BIOFILM ASSAY		VIRULENCE FACTORS Regulated by QS		Ref.
	Total Protein Content	Metabolic Activity of Biofilm-Forming by MTT Assay	Hemolysin	Protease	
Myricetin	↓(200 µg/mL)		↓(200 µg/mL)		[89]
Farrerol			↓(0.5 μg/µg/mL)	↓(0.5 μg/µg/mL)	[91]
Isorhamnetin			↓(16 µg/mL)		[92]
Lysionotin			↓(8 μg/mL)		[93]
Diosmetin			↓(32 µg/mL)		[94]
5,6,7,3′,4′-Pentahydroxyflavone	↓(80 μg/disc)				[102]
3′,4′,5-trihydroxy-6,7-dimethoxy-flavone	↓(80 μg/disc)				[102]
Phloretin			↓(16 µg/mL)		[104]
Apigenin			↓(8 µg/mL)		[95]
Epigallocatechin gallate			↓96.6% (32 µg/mL)		[96]
Baicalin			↓(16 µg/mL)		[97]
Oroxylin A			↓(8 µg/mL)		[98]
Quercetin			↓(16 µg/mL)		[90]
Dracorhodin Perochlorate			↓(16 µg/mL)		[99]
Silibinin			↓(32 µg/mL)		[100]
Baicalein	↓(32 µg/mL, 64 µg/mL)		↓(32 µg/mL, 64 µg/mL)		[88]
Baicalin			↓(16 μg/mL Baicalin with Osthole)		[105]
Rutin	↓19–88% MRSA ↓24–58%, 19–77%, 63–88% NSA-02,-06,-08 (75–600 μg/mL)	↓18–65%, 39–90%, 58–92% NSA-02,-06,-08 (75–600 µg/mL)			[101]
Chalcone	↓(76 μg/mL)		↓(38 μg/mL)		[106]

^1^ Down arrow (↓) indicates decrease of tested activity with respect to the control (ctr). Tested concentrations are reported in parenthesis. White box shows a not performed assay.

**Table 11:** Phenolic compounds inhibiting *S. aureus* biofilm formation and production of virulence Figure 1.

Compounds	BIOFILM ASSAY				VIRULENCE FACTORS Regulated by QS	Ref.
	Total Protein Content	Cristal Violet (CV) Staining	Metabolic Activity of Biofilm-Forming by MTT Assay	EPS Production	Hemolysin	
Gallic acid (GA) Ferulic acid (FA)		↓90% GA ↓7% FA		↓70% GA ↓6% FA		[71]
Eugenyl acetate					↓(150 µg/mL)	[81]
Resveratrol (Res)	↓39.8% (100 µg/mL)			↓55.4% Res + Van ↓23.4% Res	↓ (64 µg/mL)	[85]
Dihydroxybenzofurane (DHBF);Pro-antocyanidin A2 (proAc)	↓DHBF (8.2 μM) ↓proAc (6.9 μM)					[86]
Dihydroxybenzofurane (DHBF);Pro-antocyanidin A2 (proAc)	↓DHBF (8.2 μM) ↓proAc (6.9 μM)					[86]
Curcumin					↓(16 μg/mL)	[108]
Osthole					↓(at 16 μg/mL, alone and with Baicalin)	[105]
Brazilin	↓(32 μg/mL)					[109]
Punicagalin	↓47% (3.9 μg/mL); ↓90% (7.8 μg/mL).				↓(0.125 mg/mL)	[112,113]
1,2,3,4,6-Penta-O-galloyl- -D-glucopyranose (PGG)	↓93%, 96%, 97% (6.25, 12.5, 25 μM); ↓83%, 97% (50 μM)		↓7%, 58%, 87% (3.13, 12.5, 50 μM)			[115]
Rhein (Rhe) and Aloeemodin (Alo)	↓20.0% Rhe; ↓33.3% Alo					[117]
Hamamelitannin					↓(50 µg/mL)	[47,56]
Rhodomyrtone	↓(0.125–1 μg/mL)			↓(4–16 μg/mL)		[118]

^1^ Down arrow (↓) indicates decrease of tested activity with respect to the control (ctr). Tested concentrations are reported in parenthesis. White box shows a not performed assay. Vancomycin (Van)
